# Evidence and controversies in management of thyroglossal duct cyst carcinoma

**DOI:** 10.1097/MOO.0000000000000699

**Published:** 2021-01-18

**Authors:** Davide Lancini, Davide Lombardi, Cesare Piazza

**Affiliations:** a Department of Otorhinolaryngology – Head and Neck Surgery, ASST Spedali Civili of Brescia; b Department of Medical and Surgical Specialties, Radiological Sciences, and Public Health, University of Brescia, Brescia, Italy

**Keywords:** airway resection, Sistrunk procedure, thyroglossal duct cyst carcinoma, thyroid carcinoma

## Abstract

The aim of this study was to analyse the rational of the possible therapeutic approaches to thyroglossal duct cyst carcinomas (TGDCCa), especially in consideration of their potential airway involvement, discussing the most debated issues concerning employment of thyroidectomy, neck dissection and adjuvant treatments.

**Recent findings:**

The literature is unanimous in defining the Sistrunk procedure as the baseline of surgical treatment of TGDCCa, and in equating the vast majority of thyroid-like TGDCCas to classic thyroid cancers from a biological point of view, while the rarer squamous cell carcinomas seem to behave more aggressively. Thyroidectomy, neck dissection and radioactive iodine treatment are considered for high-risk lesions, with the addition of customized partial resection of laryngeal cartilages when airway involvement is encountered. Furthermore, the analysis of thyroid mutational markers has promise for accurate prevision of more aggressive clinical behaviours.

**Summary:**

Even if rare, clinicians should be aware of TGDCCa due to the possibility of incidental diagnosis and, in the case of more advanced clinical scenarios, for its potential airway involvement. Sistrunk procedure combined with thyroidectomy, neck dissection and adjuvant therapy provide excellent results in high-risk patients. Additional study of pathological thyroid markers in TGDCCa is desirable to allow more individualized treatments.

## INTRODUCTION

Thyroglossal duct cyst (TGDC) is the most common congenital lesion of the neck, usually presenting as a benign cystic mass (even though it can also variably manifest with a sinus, fistula or solid nodule) in the anterior cervical triangle of children to be managed by the well known Sistrunk procedure. When considered together with its appearance in adulthood (mostly occurring after the age of 30), it is reported in about 7% of the general population [1]. As is known, formation of TGDC is due to failure of involution (usually completed by the 10th week of development) of the endodermal invagination which during embryogenesis carries the thyroid gland from its origin at the level of the foramen cecum (floor of the primordial pharynx), through the mesoderm eventually forming the hyoid bone, to its definitive anatomical position in front of the cricoid cartilage and first tracheal rings (7th week of intrauterine development).

TGDC usually contains stratified epithelial squamous cells, pseudostratified columnar respiratory epithelium and follicular cells, all potentially giving rise to the formation of malignant neoplasms called TGDC carcinomas (TGDCCa) [2]. Their occurrence, however, is quite rare, accounting for 1–3% of all TGDC, depending on the case series and reviews considered, with a mean age at diagnosis of 39.5 years, and a prevalence that is 2.9 times higher in women [3–5].

It appears clear that rational treatment for TGDCCa should entail at least its radical surgical removal by Sistrunk procedure, as it should be always done even in cases of standalone TGDC. By contrast, the associated employment of total thyroidectomy, central and lateral neck dissections, potential resection of adjacent parts of the laryngeal skeleton and airway, and use of postoperative adjuvant radioactive iodine (RAI) or radiotherapy, are still matters of debate. The lack of a consensus on the overall management of TGDCCa clearly comes from the rarity of the lesion, with mostly case reports or case series with limited number of patients in the literature. The aim of the present review is to summarize the most common treatment strategies and the most recent advances in the management of TGDCCa, highlighting evidence-based arguments as opposed to those still mainly hypothetical. 

**Box 1:**
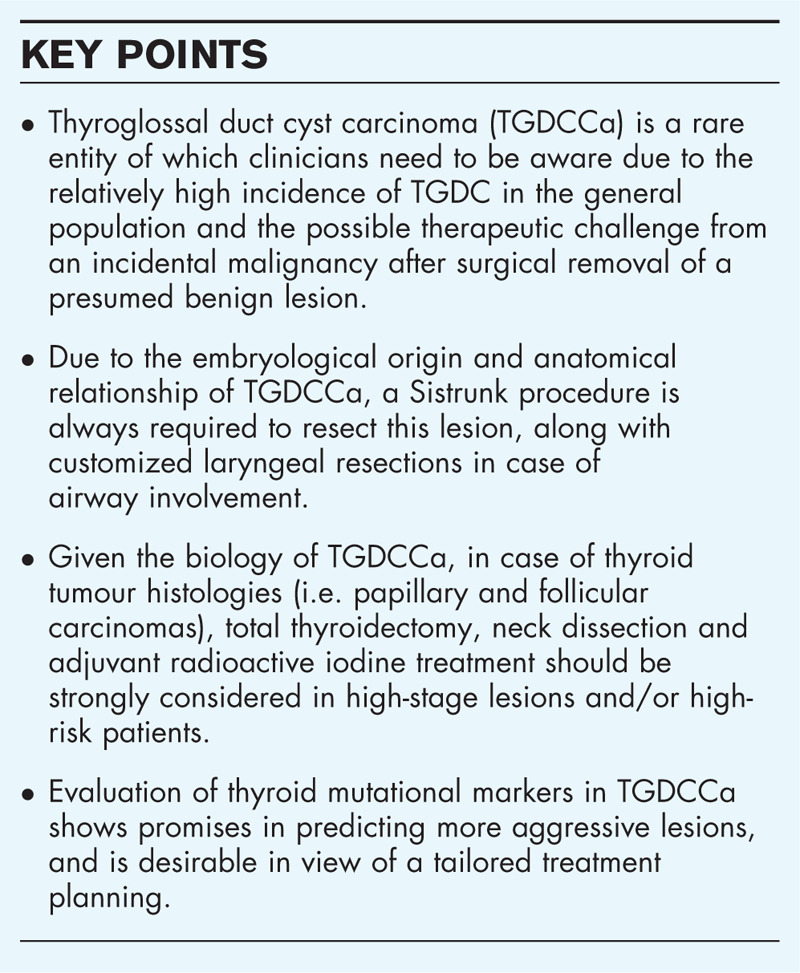
no caption available

## HISTOPATHOLOGY

TGDCCa can originate from the thyroid tissue of the cyst, as a thyroid-like neoplasm (described in about 95% of cases). The most frequently diagnosed histotype among TGDCCa is papillary thyroid cancer (PTC – in >90% of cases), followed by other usual thyroid histologies such as follicular carcinoma [6,7]. The current literature is, however, unanimous in defining thyroid-like TGDCCa as a neoplasm arising *de novo* in a TGDC, and not as a metastasis from an underlying primary lesion of the thyroid [8]. Such a ‘*de novo* hypothesis’ has been indirectly confirmed by Rossi *et al*. [9] who demonstrated the lack of both morphological and molecular concordance between TGDCCa and concomitant thyroid cancers when present.

Even if much more rarely (<5% of cases), TGDCCa can also originate from the cystic epithelial lining as a squamous cell carcinoma (SCC). These neoplasms tend to behave more aggressively than their PTC or follicular counterparts and usually present a more dismal prognosis. Very seldom, SCC and thyroid-like tumours may be synchronously present within the same TGDC, with only four cases reported in the literature to date [10–13].

Due to their different embryological origin, parafollicular or C cells (as part of the neuroendocrine system and therefore deriving from neural crest cells invading the ultimobranchial bodies, which further differentiate after fusion with the median anlage) were not found in a 125 histopathological analysis of TGDCs with that aim in mind [14^▪^]. As a consequence, no case of medullary thyroid carcinoma (MTC) has been reported to arise in a TGDC [15] and, additionally, as a further confirmation of the abovementioned ‘*de novo* hypothesis’, no metastasis to the TGDC from MTC has been ever described. However, an interesting report from Keelawat and Bychkov [14^▪^] recently reported identification of neuroendocrine cells positive for calcitonin, chromogranin A and synaptophysin (but negative for the carcinoembryonic antigen, which is in turn positive in the typical immunophenotype of C cells), not in the thyroid remnant of TGDC, but within the respiratory epithelium lining the inner surface of the cyst itself. These calcitonin-positive neuroendocrine cells of the TGDC lining could be a potential source of rare neuroendocrine tumours (not to be confounded with MTC), previously described in foregut derivatives of pancreatic and respiratory origin, and possibly reported in one case of TGDCCa (Mousavi Z, Mashad University of Medical Sciences, Iran; personal communication to be found at https://goo.gl/aqw9Rd).

## CLINICAL PRESENTATION AND DIAGNOSTIC WORK-UP

More than 75% of TGDCCa are incidentally diagnosed at definitive histopathology after removal of an otherwise normal and apparently benign TGDC, being the mean tumour size found within the cyst of 17.2 mm [4]. In the remaining cases, preoperative suspicion of malignancy is raised by clinical and/or radiological findings. In particular, a midline, growing, firm, solid nodule with irregular borders in the median region of the neck of an adult patient (quite rarely a child), not sliding on the thyroid cartilage or hyoid bone, and not moving synchronously with the tongue extrusion, sometimes causing pain and dysphagia, may help in differentiating a TGDCCa from a common TGDC or other benign conditions. Concurrent lymph node swelling can add to further clinical suspicion.

Regardless of clinical presentation, careful neck ultrasonography is always mandatory in order to evaluate the morphology of the nodule, presence of suspicious features (such as a prominent solid part, microcalcifications, asymmetric wall thickening, hypervascularization) and assess the presence and features of the thyroid gland, excluding other ectopic localizations, as well as lymph node metastases [5,16]. Moreover, when suspicious features are found at ultrasonography, the procedure can be supplemented with ultrasonography-guided fine needle aspiration cytology (FNAC) [5,17].

FNAC has shown robust results, reaching values of sensitivity and specificity up to 100%, especially when performed with the cooperation of dedicated radiologists and cytologists, directing their sample to the solid part of the lesion itself, usually represented by its wall [18]. In fact, such a trick may greatly help in reducing the false-negative rate from dilution of the cytologic population by the cystic content. Moreover, repeated FNAC of any residual mass after aspiration of the cyst content may yield more diagnostic results. Due to the rarity of TGDCCa in the paediatric population, FNAC might be carried out only if suspicion is raised by ultrasonography [19]. By contrast, due to low cost and high accuracy guaranteed by FNAC, different authors recommend its routine use in all TGDC diagnosed or treated in the adult population [5,18]. When features suspicious for malignancy are found at the first investigation, magnetic resonance (MR) and/or computed tomography (CT) are required to assess the relationships of the TGDCCa with prelaryngeal muscles, hyoid bone, and, especially, thyroid and cricoid cartilages, and to evaluate nodal status, if not already documented by ultrasonography.

In 1976, Widstrom *et al.*[20] defined the diagnostic criteria for definition of a TGDCCa: localization of the carcinoma to a clearly demonstrable TGDC or to the anterior midline from the base of the tongue to the thyroid isthmus through the hyoid bone (while carcinoma in a lingual thyroid should be excluded from such a diagnosis when no descent at all occurred); and no carcinoma on histopathological examination of the thyroid gland. It is therefore fundamental to correctly estimate the pertinence and primary nature of the neoplasm, in order to preoperatively differentiate the lesion from a cystic metastasis of a tumour originating in other head and neck sites while, on the other hand, due to the high chance of a multifocal thyroid cancer, the presence of a synchronous thyroid malignancy might not be sufficient to exclude a primary origin from TGDC.

## SURGICAL TREATMENT OF THE PRIMARY LESION

For the abovementioned embryological reasons, Sistrunk procedure is the minimum surgical procedure required for appropriate management of a TGDCCa [21]. In confirmation of this, Patel *et al.*[7] reviewed the outcomes in 62 patients treated for TGDCCa: at univariate analysis, the only prognostic factor affecting overall survival was the extent of surgery employed for removal of the primary lesion (Sistrunk procedure or less than that), while the addition of thyroidectomy or neck dissection did not show any influence on survival outcomes.

**FIGURE 1:**
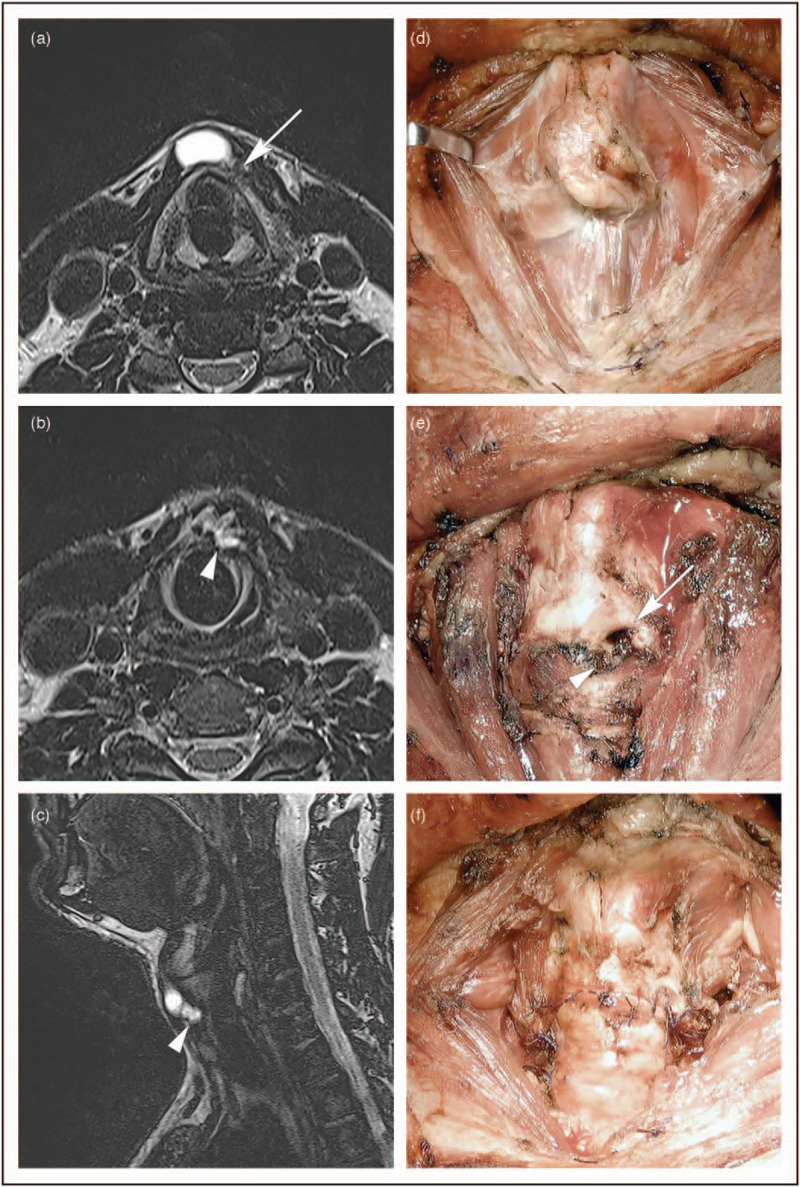
Fifty-one-year-old man complaining of a slowly growing, hard mass in the anterior midline of the neck for 6 months. Clinical examination documented a solid nodule, firmly attached to the thyroid and cricoid cartilages, 3 cm in diameter. Neck ultrasonography showed the presence of a cystic lesion, consistent with a TGDC, with an inferior solid component. No pathological neck lymph node was detected and a normal thyroid gland was found in its physiological position. The FNAC of the solid part of the TGDC was suspicious for PTC (TGDCCa). The MR T2-weighted axial sequences showed a cystic lesion, with a solid vascularized portion infiltrating the paramedian inferior edge of the left thyroid lamina (white arrow) (a), the cricoid-thyroid membrane and the superior aspect of the cricoid arch (white arrowhead) (b). A T2-weighted sagittal left paramedian MR sequence confirmed the presence of a cystic lesion superiorly, with an inferior solid TGDCCa component infiltrating the crico-thyroid membrane (white arrowhead) (c). The patient underwent surgical removal of the lesion by Sistrunk procedure, total thyroidectomy with central compartment neck dissection, drilling of the inferior part of the left thyroid lamina, resection of the cricoid arch and subsequent thyro-crico-tracheal anastomosis (d--f). Thyroid cartilage erosion, crico-thyroid membrane infiltration, and involvement of the superior edge of the cricoid arch and left crico-thyroid muscle were intraoperatively confirmed (e). Final histopathological examination reported PTC arising from the TGDC, with invasion of the cricoid-thyroid membrane and superior aspect of the cricoid arch (pT4), but no disease in the thyroid gland or central compartment lymph nodes. The patient was extubated the day of surgery without tracheostomy or naso-gastric feeding tube, started a soft oral diet the day after surgery and a normal diet 2 days later. He was discharged from the hospital at 10 days after surgery and underwent adjuvant RAI. He is without evidence of disease at 7 months after surgery.

Due to the close proximity of these lesions to the laryngeal axis, it is not rare to find descriptions of laryngeal involvement with extension of a huge benign TGDC through the thyrohyoid membrane into the preepiglottic space, presenting as a ‘dumbbell-shaped’ lesion potentially obliterating the supraglottic airway [22^▪^]. In case of laryngeal framework erosion/infiltration by TGDCCa (described in four cases affecting the thyroid, two the cricoid, two the thyrohyoid and one the crico-thyroid membranes) [22^▪^], its surgical excision must include some type of partial resection of laryngeal cartilages in order to completely remove the lesion within free margins and, simultaneously, maintain satisfactory laryngeal functions. In this sense, a case of limited crico-tracheal resection with subsequent thyro-crico-tracheal anastomosis treated at our Institution is briefly described (Fig. 1). Even though limited to anecdotal experience, circumferential airway resection with end-to-end anastomosis seems amply justified according to the principles of surgical radicality followed for thyroid cancers infiltrating the airway. In such a case, indeed, whenever an airway framework invasion exceeding the external perichondrium is detected (Shin II or more according to the classification by Shin *et al*.) [23], its complete resection with ensuing direct end-to-end anastomosis allows for adequate oncological radicality and overall optimal functional preservation [24–26].

The current literature tends to compare common PTC with TGDCCa from biologic and oncologic points of view in order to better define and plan the extent of surgery, need for adjuvant treatment and stratify different risk categories [3,4,27,28]. However, even if it seems a logical choice according to histological and pathological features, it has to be clearly kept in mind that, arising from a TGDC and not from an entire thyroid gland, the transition from a low-T category lesion (when the nodule is completely surrounded by an intact epithelial lining) to a ‘virtual’ T3b-T4a, with extracystic growth and airway infiltration, is definitively more straightforward and rapid. In addition, in this sense, possible lymph nodes metastases to the central compartment of the neck must be always ruled out and, in case of high-T category TGDCCa, a prophylactic level VI neck dissection usually seems appropriate.

## MANAGEMENT OF THE THYROID GLAND

When preoperative neck ultrasonography shows a synchronous lesion within the thyroid gland, the decision to proceed with simultaneous thyroidectomy is beyond any question. By contrast, if the thyroid gland does not appear to be involved at preoperative examination or the TGDCCa is incidentally discovered at final histopathology, second-look completion of Sistrunk procedure with other procedures is questionable [4]. Referring to thyroid oncology, several authors [6–8] categorize patients into low-risk and high-risk groups. Patients younger than 45 years, with a tumour size (not TGDC size) less than 1.5 cm, without extracystic extension and no loco-regional or distant localizations at ultrasonography or other imaging, seem manageable by Sistrunk procedure alone. Otherwise, more aggressive surgical treatment is warranted [4,5]. In any case, if, on one hand, thyroidectomy may increase the risk of recurrent laryngeal nerve(s) injury and transient/permanent hypocalcemia, on the other hand, the addition of thyroidectomy to Sistrunk procedure will allow better staging, completion of treatment with adjuvant RAI therapy and easier follow-up by both ultrasonography and thyroglobulin assay.

When total thyroidectomy has been accomplished with a radicalization intent, the tumour was found in about 35% of cases, which is similar to the incidental finding of thyroid tumours by autoptic studies on the general population [29]. By contrast, Hartl *et al.*[28] and Bakkar *et al.*[30^▪▪^] reported evidence of synchronous thyroid tumours in 56 and 62% of their cases, respectively, demonstrating that TGDCCa is very similar to PTC and may present a high rate of multifocality.

Interestingly, Bakkar *et al.*[30^▪▪^] recently assessed the utility of mutational markers in guiding the preoperative surgical decision process by exploring the potential correlation between BRAF^V600E^ positivity and the local behaviour of the TGDCCa. In particular, these authors found a significant and strong association between BRAF^V600E^ mutation positivity and high-T category (pT3 for microscopic extra-cystic soft tissues involvement), thus requiring total thyroidectomy and postoperative adjuvant RAI. These data, independently confirmed by Rossi *et al*. [9], who found synchronous thyroid and TGDCCa in 80% of BRAF^V600E^ positive patients, would suggest the opportunity to assess the BRAF^V600E^ mutational status in every TGDCCa by preoperative FNAC. This would allow stratifying patients into those with a potentially advanced and aggressive disease (BRAF^V600E^ positive), to be straightforward submitted to concomitant Sistrunk procedure and total thyroidectomy with postoperative RAI, and those who can be initially managed in a more conservative way by standalone Sistrunk procedure, as they are BRAF^V600E^ negative.

## MANAGEMENT OF CERVICAL LYMPH NODES

Positive lymph node rates in past studies were reported to be between 7 and 15%, while a recent meta-analysis reported a pooled rate of 16.4% [4]. Hartl *et al.*[28], who found a higher rate of tumour multifocality, also indicated a 75% rate of lymph nodes metastases: 40% in the central compartment and 60% in the lateral neck. The authors stated that the presence of tumour in the thyroid gland did not correlate with nodal status, as half of patients with lymph node metastases did not show any pathologic thyroid gland involvement. These data further confirm the similar biological behaviour of TGDCCa and PTC. Of note, the authors underline the possibility, in around 40% of patients, to have ‘skip’ metastases to the lateral neck (levels III and IV) without central compartment involvement. This can be possibly related to the peculiar anatomic location of TGDCCa whose lymphatic drainage may primarily follow the superior thyroid pedicle to the jugular lymphatic chain.

In their meta-analysis, Rayess *et al.*[4] estimated a recurrence rate of 4.3%, which, in 42.9% was at the level of the lymph nodes basin. In our opinion, similar to what generally suggested for common PTC, neck dissection might be performed when there is preoperative evidence of nodal involvement at ultrasonography, or following an intraoperative findings of enlarged lymph nodes. For large TGDCCa, in close contact with the thyroid gland and extracystic spread to the surrounding soft tissues (T3) or macroscopic evidence of airway involvement (T4), a central compartment exploration and dissection might be required, together with thyroidectomy and airway resection if needed.

When dealing with TGDCCa SCC, due to its worse prognosis and more aggressive behaviour [5], central compartment and level IA dissection may be helpful to better stage the disease and possibly indicate the need for adjuvant radiotherapy on the lateral neck levels [31].

## POSTOPERATIVE TREATMENTS

Use of adjuvant RAI after TGDCCa excision follows the guidelines commonly applied for treatment of PTC, being indicated in case of extension to the surrounding tissues, multifocality, involvement of the thyroid gland and lymph nodes, or in the presence of aggressive histological variants [32,33].

In case of the much rarer TGDCCa with SCC histology, use of adjuvant radiotherapy should be evaluated only for involvement of adjacent structures, positive surgical margins, multiple positive lymph nodes and nodal extracapsular extension. As for RAI, due to the extreme rarity of such lesions, therapeutic guidelines are therefore substantially in line with those of more common SCC at other head and neck sites.

## ONCOLOGIC OUTCOMES

Although TGDCCa is thought to be associated with good survival, with a long-term disease-specific mortality close to zero similar to what observed for PTC normally arising within the thyroid lobes, there are limited data on detailed oncologic outcomes. The only systematic review to date [4] seems to confirm that TGDCCa is a relatively indolent disease, mostly associated with good prognosis, with only 4.3% of patients showing recurrence, mainly at the level of the neck basins (43%, none of whom received prior neck dissection), within a follow-up time of 42.1 months. All patients with recurrent disease had PTC histology and only one died due to distant lung and brain metastases at 35 months after diagnosis and treatment (Sistrunk procedure and thyroidectomy followed by RAI).

The longest follow-up (median, 71 months; range, 1–456) was reported by Patel *et al.*[7] in a pooled-analysis including five cases managed at the Memorial Sloan-Kettering Cancer Center and 57 taken from the literature. In line with the oncologic outcomes observed in PTC, the authors reported 5 and 10-year overall survival rates of 100 and 95.6%, respectively.

## CONCLUSION

TGDCCa represents a rare entity that needs to be recognized due to the high overall incidence of benign TGDC in the general population. Moreover, the embryological origin and anatomical location of these tumours render them at risk of infiltration of the laryngeal muscles, cartilages and upper airways, as described herein. The current management of TGDCCa with Sistrunk procedure, thyroidectomy, neck dissection and RAI for high-risk patients has proven sound with long-term survival rates that exceed 95%. Further study will be helpful in applying new thyroid biological and molecular markers in the evaluation of such a rare disease, allowing, when possible, more customized and conservative therapeutic approaches.

## Acknowledgements

*None.*

### Financial support and sponsorship

*None.*

### Conflicts of interest

*There are no conflicts of interest.*

## REFERENCES AND RECOMMENDED READING

Papers of particular interest, published within the annual period of review, have been highlighted as:

▪ of special interest
▪▪ of outstanding interest
